# Supplementary material for: Host Factors Affect the Gut Microbiome More Significantly than Diet Shift
Source: Microorganisms. 2021 Dec 6;9(12):2520. doi: 10.3390/microorganisms9122520 (PMC8707884; doi:10.3390/microorganisms9122520)
Supplement: Supplementary file 1 [file microorganisms-09-02520-s001.zip › microorganisms-1433783-supplementary.pdf]

SUPPLEMENTAL INFORMATION TITLES AND LEGENDS

Supplemental Figures

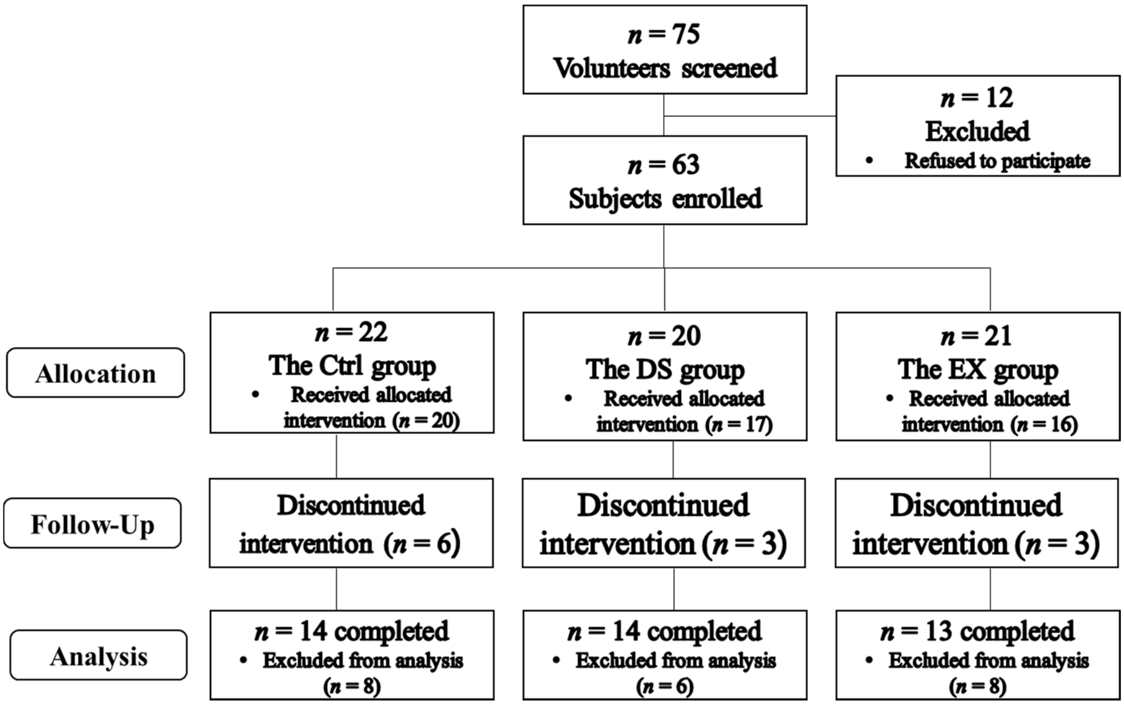

Figure S1. Flow chart for the study subjects. The Ctrl, DS, and EX represents the control, diet shift, and exercise groups each.

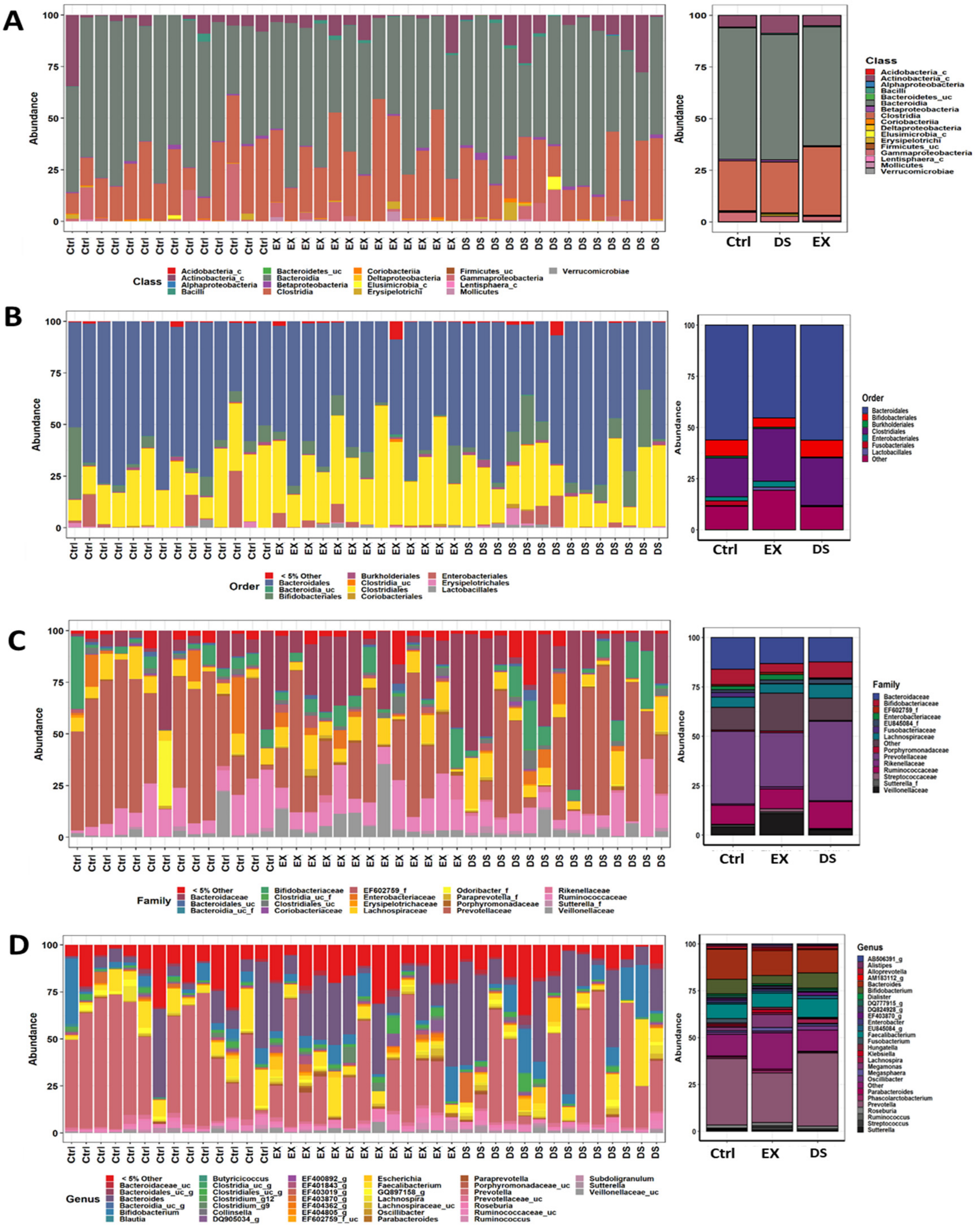

Figure S2. The composition changes of gut microbiome after diet shift or exercise. The composition changes of gut microbiome at (A) class levels (B) order levels (C) family levels and (D) genus during the 12 week experimental period are shown. The Ctrl, EX, and DS represents the control, exercise, and diet shift groups each.

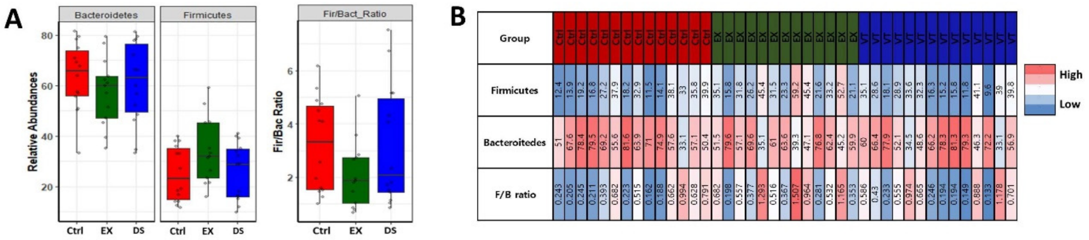

Figure S3. The abundance change of Bacteroitedes and Firmicutes in the gut microbiome in the three experimental groups. The Ctrl, EX and DS represents the control, exercise, and diet shift groups each. .

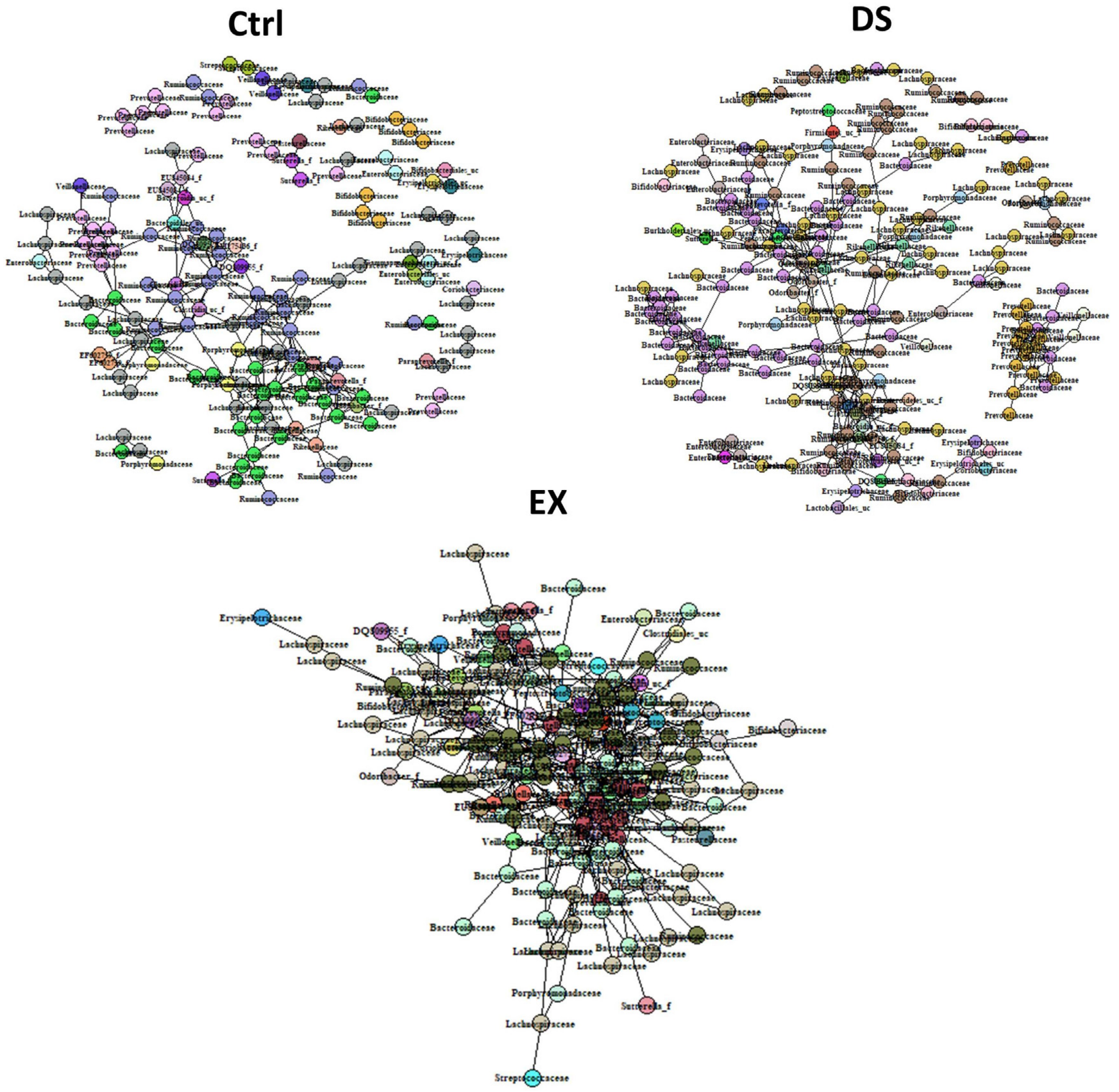

Figure S4. Co-occurrence network analysis by the ReBoot algorithm for the Ctrl, EX, and DS groups. Color-coded network graph representations of the co-occurrence interactions among OTUs in family level.

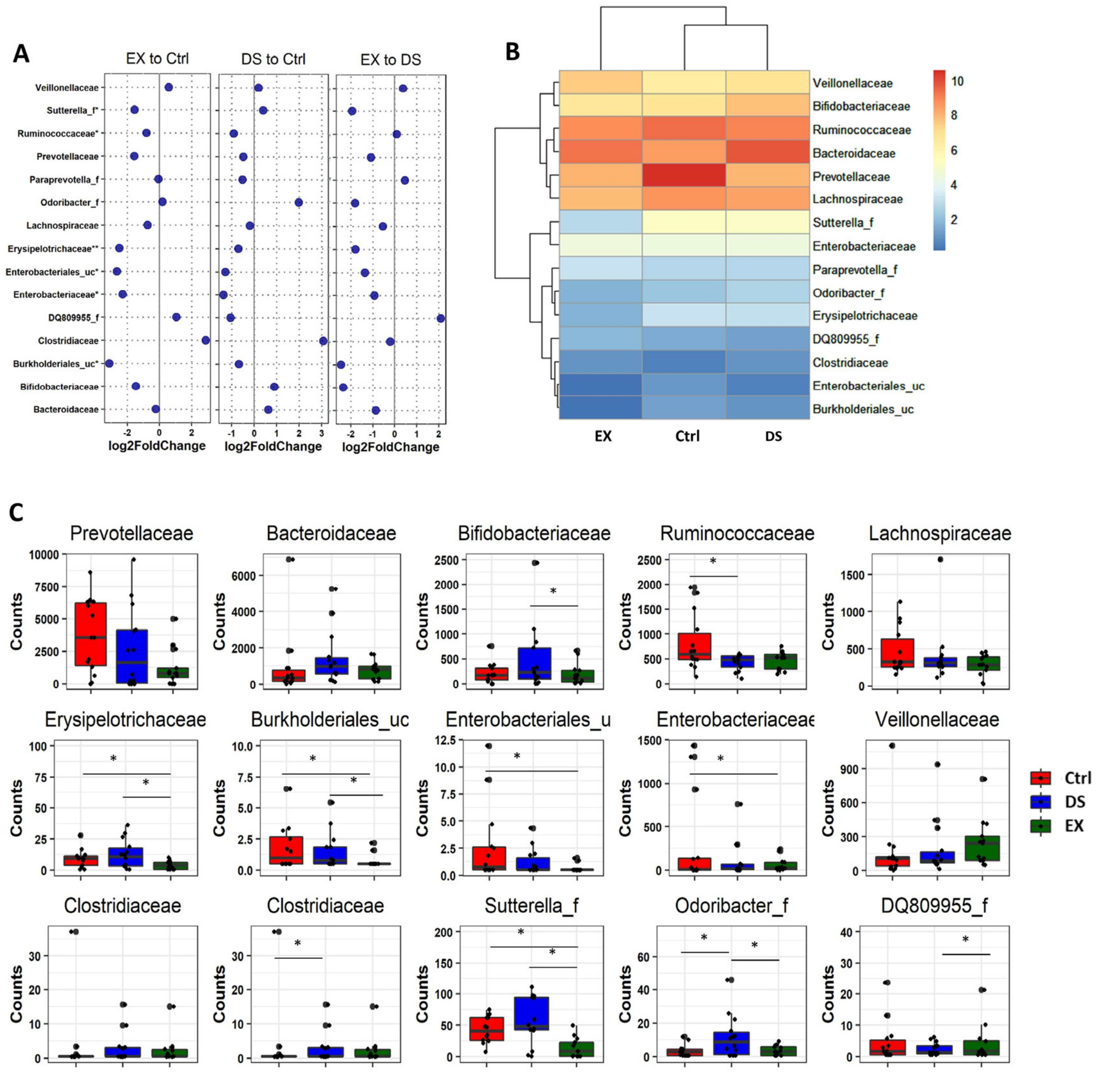

Figure S5. The most signficantly changed microbes at family level by differential abundance analysis. (A) Log2 fold change of abundance on most abundantly present families in the gut microbiome of the three experimental groups analyzed by DESeq2 differential abundance analysis. Each point represents a species comparison between two experimental groups. (B) Heatmap of most abundantly present species in the three experimental groups. (C) Normalized abundances of significantly different 15 families of interest that were identified from differential abundance analyses. Boxplot represent normalized count abundances of individual species in each group. *p* value < 0.05 was considered as significant. The Ctrl, EX, and DS represents the control, exercise, and diet shift groups each.

Table S1. The valid reads of 16S rRNA amplicon sequence.

Group	Ctrl	EX	DS
**Total reads**	7664±2045	3275±165	8928±1198

All values are the mean±SEM. OTUs, operational taxonomic units; Ctrl, the control group; Ex, the physical exercise group; DS, the vegetarian diet group.

Table S2. The α-diversity indexes for each sample. *p* value calculated by Kruskal_Wallis test, and Wilcoxon sum test was used for group comparison**.** The Ctrl, EX, and DS represents the control, exercise, and diet shift groups each.

Group	ACE	se.ACE	Shannon	Simpson	InvSimpson	Fisher	Evenness
**Ctrl**	254.94	8.30	2.86	0.85	6.47	35.84	0.52
**Ctrl**	288.78	8.82	3.15	0.88	8.39	40.23	0.56
**Ctrl**	399.07	10.02	3.28	0.90	9.74	51.29	0.55
**Ctrl**	290.28	9.13	2.79	0.88	8.25	30.74	0.49
**Ctrl**	252.85	8.32	3.37	0.93	13.35	33.90	0.61
**Ctrl**	245.04	8.17	3.81	0.96	22.34	41.74	0.69
**Ctrl**	277.56	8.90	2.90	0.86	7.18	30.34	0.52
**Ctrl**	269.96	8.44	3.92	0.96	26.14	46.38	0.70
**Ctrl**	299.64	9.68	3.61	0.94	15.92	46.12	0.63
**Ctrl**	274.45	8.98	3.54	0.93	15.35	41.76	0.63
**Ctrl**	215.64	7.78	3.62	0.94	15.60	35.85	0.67
**Ctrl**	343.96	10.05	4.18	0.97	28.66	64.01	0.72
**Ctrl**	242.89	8.82	3.40	0.94	15.76	36.67	0.62
**Ctrl**	200.30	7.47	3.51	0.94	16.64	30.61	0.66
**EX**	260.31	8.31	3.96	0.96	24.44	44.25	0.71
**EX**	230.76	7.99	2.72	0.79	4.76	33.03	0.50
**EX**	297.10	8.81	4.41	0.98	49.36	58.50	0.78
**EX**	256.62	7.97	4.02	0.96	25.09	50.27	0.72
**EX**	234.80	7.82	3.91	0.96	26.89	42.36	0.72
**EX**	142.47	6.37	3.69	0.96	23.16	33.02	0.74
**EX**	244.33	8.84	3.67	0.95	19.10	37.02	0.67
**EX**	160.22	6.29	3.18	0.91	10.58	26.01	0.63
**EX**	309.78	9.48	4.18	0.97	30.11	57.79	0.73
**EX**	310.26	9.54	3.59	0.93	13.72	44.36	0.63
**EX**	282.15	9.84	3.87	0.95	21.34	43.65	0.69
**EX**	314.10	9.22	4.32	0.97	37.29	63.44	0.75
**EX**	240.73	9.00	3.15	0.91	10.59	31.20	0.57
**DS**	353.45	9.95	3.61	0.94	16.56	47.48	0.62
**DS**	350.40	9.45	4.07	0.97	29.45	53.76	0.69
**DS**	399.62	10.38	3.37	0.89	8.96	52.12	0.56
**DS**	349.99	10.12	3.80	0.95	19.08	52.27	0.65
**DS**	341.50	9.78	3.90	0.95	21.72	55.46	0.67
**DS**	311.02	9.07	3.75	0.95	19.72	40.71	0.65
**DS**	405.98	10.70	3.66	0.95	19.66	54.19	0.61
**DS**	157.14	6.57	3.08	0.90	9.89	25.21	0.61
**DS**	350.53	9.47	3.14	0.87	7.73	46.25	0.54
**DS**	223.13	7.43	3.21	0.90	10.45	31.11	0.59
**DS**	311.94	8.91	3.97	0.96	26.27	53.19	0.69
**DS**	192.07	7.62	2.77	0.84	6.20	24.17	0.53
**DS**	153.86	7.20	2.47	0.86	7.31	15.31	0.49
**DS**	406.30	10.47	3.93	0.95	19.89	60.14	0.65
**Kruskal-Wallis *p* value**	0.09	0.28	0.15	0.10	0.10	0.47	**0.02**
***p* value CvsE**	0.27	0.35	0.08	0.28	0.05	0.46	**0.03**
***p* value CvsD**	0.25	0.47	0.74	0.90	0.73	0.47	0.98
***p* value DvsE**	0.06	0.18	0.17	0.34	0.09	0.97	**0.02**

Table S3. Comparison of taxonomy abundance at Phylum level. The Ctrl, EX, and DS represents the control, exercise, and diet shift groups each.

	Phylum	Ctrl (%)	DS (%)	EX (%)
1	Bacteroidetes	63.57	60.65	57.55
2	Firmicutes	25.02	26.02	34.02
3	Proteobacteria	5.21	3.77	2.48
4	Actinobacteria	5.91	8.92	5.3
5	Elusimicrobia	0.11	0.44	0
6	Tenericutes	0.11	0.18	0.6
7	Lentisphaerae	0.07	0.03	0.01
8	Verrucomicrobia	0	0	0.04
9	Acidobacteria	0	0	0.01

Table S4. Co-occurrence network indices. The Ctrl, EX, and DS represents the control, exercise, and diet shift groups each.

**Network Indexes indices**	**Ctrl**	**DS**	**EX**
Node	161	205	171
Edge	274	397	535
Network Density	0.021	0.019	0.037
Assortativity	-0.010	-0.008	-0.009
Module	28	20	8
Network Heterogeneity	0.613	0.718	0.676
Network Centralization	0.11	0.071	0.084
Clustering coefficient	0.424	0.336	0.347
Average Number of Neighbors	4.465	4.09	4.693

Table S5. The comparison of the abundance of the phyla constituting the gut microbiome of the three experimental groups by DESeq2 differential abundance analysis. The Ctrl, EX, and DS represents the control, exercise, and diet shift groups each.

DS to Ctrl

	Phylum	baseMean	log2FoldChange	lfcSE	stat	pvalue	padj
6	Tenericutes	13.522	-0.544	1.414	-0.385	0.700	0.909
8	Verrucomicrobia	0.637	-0.453	3.572	-0.127	0.899	0.909
4	Actinobacteria	316.759	-0.385	0.616	-0.624	0.532	0.909
3	Firmicutes	1471.933	0.187	0.267	0.700	0.484	0.909
2	Bacteroidetes	3454.093	0.347	0.295	1.173	0.241	0.909
9	Elusimicrobia	0.017	0.408	3.573	0.114	0.909	0.909
7	Acidobacteria	0.112	0.869	3.573	0.243	0.808	0.909
5	Lentisphaerae	1.018	0.966	2.410	0.401	0.689	0.909
1	Proteobacteria	152.004	1.304	0.546	2.387	0.017	0.153

EX to Ctrl

	Phylum	baseMean	log2FoldChange	lfcSE	stat	pvalue	padj
5	Verrucomicrobia	0.637	-3.529	3.620	-0.975	0.330	0.593
3	Tenericutes	13.522	-2.393	1.441	-1.661	0.097	0.290
8	Acidobacteria	0.112	-1.357	3.641	-0.373	0.709	0.776
9	Elusimicrobia	0.017	-1.034	3.642	-0.284	0.776	0.776
2	Firmicutes	1471.933	-0.468	0.273	-1.716	0.086	0.290
7	Actinobacteria	316.759	0.270	0.629	0.429	0.668	0.776
4	Bacteroidetes	3454.093	0.369	0.301	1.225	0.220	0.496
1	Proteobacteria	152.004	1.467	0.559	2.624	0.009	0.078
6	Lentisphaerae	1.018	1.894	2.496	0.758	0.448	0.672

EX to DS

	Phylum	baseMean	log2FoldChange	lfcSE	stat	pvalue	padj
7	Lentisphaerae	1.018	-0.927	2.501	-0.371	0.711	0.866
3	Actinobacteria	316.759	-0.655	0.629	-1.041	0.298	0.866
8	Proteobacteria	152.004	-0.164	0.559	-0.292	0.770	0.866
9	Bacteroidetes	3454.093	-0.023	0.301	-0.075	0.940	0.940
1	Firmicutes	1471.933	0.655	0.273	2.402	0.016	0.147
6	Elusimicrobia	0.017	1.443	3.643	0.396	0.692	0.866
2	Tenericutes	13.522	1.849	1.435	1.288	0.198	0.866
5	Acidobacteria	0.112	2.226	3.642	0.611	0.541	0.866
4	Verrucomicrobia	0.637	3.076	3.619	0.850	0.395	0.866

Table S6. The comparison of the abundance of the families constituting the gut microbiome of the three experimental groups by DESeq2 differential abundance analysis. The Ctrl, EX, and DS represents the control, exercise, and diet shift groups each.

	Family	baseMean	EX to DS				DS to Ctrl				EX to Ctrl			
			log2Fold Change	lfcSE	stat	pvalue	log2Fold Change	lfcSE	stat	pvalue	log2Fold Change	lfcSE	stat	pvalue
1	Prevotellaceae	2556.55	-1.09	1.01	-1.07	0.28	-0.48	0.99	-0.48	0.63	-1.57	1.01	-1.54	0.12
2	Bacteroidaceae	1053.93	-0.86	0.59	-1.45	0.15	0.63	0.58	1.09	0.28	-0.23	0.59	-0.38	0.70
3	Bifidobacteriaceae	576.11	-2.34	0.86	-2.71	0.01	0.90	0.85	1.06	0.29	-1.45	0.86	-1.68	0.09
4	Ruminococcaceae	570.65	0.09	0.31	0.30	0.76	-0.90	0.30	-2.97	0.00	-0.80	0.31	-2.61	0.01
5	Lachnospiraceae	383.23	-0.54	0.38	-1.43	0.15	-0.19	0.37	-0.50	0.61	-0.73	0.38	-1.92	0.05
6	Veillonellaceae	199.86	0.38	0.58	0.66	0.51	0.20	0.56	0.36	0.72	0.58	0.58	1.01	0.31
7	Enterobacteriaceae	153.38	-0.93	1.07	-0.86	0.39	-1.36	1.05	-1.29	0.20	-2.29	1.07	-2.13	0.03
8	Clostridiales_uc	73.09	-0.36	0.38	-0.95	0.34	0.36	0.38	0.96	0.34	0.00	0.39	0.00	1.00
9	EU845084_f	59.22	-1.38	1.71	-0.81	0.42	0.71	1.68	0.42	0.67	-0.67	1.71	-0.39	0.69
10	Bacteroidales_uc	39.95	-0.66	0.52	-1.27	0.20	0.08	0.51	0.16	0.87	-0.58	0.52	-1.11	0.27
11	Sutterella_f	37.93	-1.94	0.57	-3.41	0.00	0.40	0.55	0.73	0.46	-1.54	0.57	-2.70	0.01
12	EF602759_f	34.15	0.95	1.10	0.87	0.38	-0.78	1.08	-0.72	0.47	0.18	1.09	0.16	0.87
13	Rikenellaceae	30.83	0.49	0.98	0.50	0.62	1.18	0.96	1.22	0.22	1.67	0.98	1.70	0.09
14	Porphyromonadaceae	28.48	-0.02	0.43	-0.05	0.96	0.58	0.42	1.37	0.17	0.56	0.43	1.29	0.20
15	Paraprevotella_f	20.07	0.47	1.00	0.47	0.64	-0.52	0.98	-0.53	0.60	-0.05	1.00	-0.05	0.96
16	Coriobacteriaceae	12.75	-1.18	0.81	-1.45	0.15	0.13	0.78	0.16	0.87	-1.05	0.81	-1.29	0.20
17	Erysipelotrichaceae	11.98	-1.79	0.75	-2.38	0.02	-0.70	0.70	-1.00	0.32	-2.49	0.75	-3.32	0.00
18	Streptococcaceae	9.63	1.09	1.16	0.93	0.35	-0.04	1.15	-0.03	0.97	1.05	1.16	0.90	0.37
19	Clostridia_uc_f	9.02	-0.30	0.43	-0.70	0.49	0.03	0.42	0.08	0.94	-0.27	0.44	-0.61	0.54
20	Odoribacter_f	5.76	-1.80	0.72	-2.49	0.01	1.99	0.71	2.82	0.00	0.19	0.75	0.26	0.80
21	Pasteurellaceae	5.60	-0.61	1.29	-0.47	0.64	-0.56	1.25	-0.44	0.66	-1.16	1.29	-0.90	0.37
22	Bacteroidia_uc_f	5.43	-0.52	0.67	-0.77	0.44	-0.02	0.64	-0.03	0.98	-0.54	0.68	-0.79	0.43
23	Erysipelotrichales_uc	4.93	0.17	1.56	0.11	0.91	-2.81	1.50	-1.87	0.06	-2.64	1.53	-1.72	0.09
24	DQ809955_f	4.54	2.11	0.91	2.31	0.02	-1.04	0.91	-1.15	0.25	1.06	0.90	1.19	0.24
25	AM275436_f	4.21	0.26	1.43	0.18	0.85	1.15	1.42	0.81	0.42	1.41	1.45	0.97	0.33
26	DQ809526_f	2.09	0.41	1.06	0.39	0.70	1.22	1.09	1.11	0.27	1.63	1.11	1.47	0.14
27	Peptostreptococcaceae	1.93	0.55	1.05	0.52	0.60	1.01	1.08	0.94	0.35	1.56	1.10	1.43	0.15
28	Lactobacillaceae	1.89	1.08	1.47	0.73	0.46	-2.30	1.41	-1.63	0.10	-1.23	1.43	-0.86	0.39
29	Burkholderiales_uc	1.67	-2.44	1.16	-2.11	0.04	-0.67	1.03	-0.65	0.51	-3.12	1.15	-2.70	0.01
30	AF371945_f	1.66	0.64	1.83	0.35	0.72	1.76	1.84	0.95	0.34	2.40	1.87	1.28	0.20
31	Clostridiaceae	1.47	-0.20	1.44	-0.14	0.89	3.08	1.47	2.09	0.04	2.88	1.51	1.91	0.06
32	Mogibacterium_f	1.29	-0.11	1.25	-0.09	0.93	0.51	1.23	0.42	0.68	0.41	1.27	0.32	0.75
33	EF445272_f	1.08	1.20	2.30	0.52	0.60	-0.23	2.27	-0.10	0.92	0.97	2.31	0.42	0.67
34	Enterobacteriales_uc	1.02	-1.36	1.28	-1.06	0.29	-1.27	1.17	-1.09	0.28	-2.63	1.26	-2.09	0.04
35	AB185535_f	1.00	-1.52	3.14	-0.49	0.63	-1.72	3.04	-0.57	0.57	-3.25	3.12	-1.04	0.30

Table S7. The List of microbial families constituting the gut microbiome of the three experimental groups. The Ctrl, EX, and DS represents the control, exercise, and diet shift groups each.

	Family	Ctrl (%)	DS (%)	EX (%)
1	Prevotellaceae	46.61	30.02	28.02
2	Bacteroidaceae	11.48	25.42	23.3
3	Ruminococcaceae	12.99	11.17	13.58
4	Lachnospiraceae	6.68	8.09	8.31
5	Bifidobacteriaceae	5.63	8.59	5.08
6	Veillonellaceae	2.86	2.92	7.85
7	Enterobacteriaceae	4.31	2.41	1.92
8	Clostridiales_uc	1.15	1.83	2.13
9	EU845084_f	1.01	1.84	0.98
10	Odoribacter_f	2.3	0.23	0.08
11	Bacteroidales_uc	0.71	0.83	0.87
12	EF602759_f	0.54	0.48	1.35
13	Rikenellaceae	0.23	0.71	1.22
14	Sutterella_f	0.69	1.06	0.39
15	Porphyromonadaceae	0.33	0.61	0.91
16	Paraprevotella_f	0.25	0.38	0.68
17	Streptococcaceae	0.33	0.2	0.39
18	Erysipelotrichales_uc	0.27	0.56	0.06
19	Coriobacteriaceae	0.24	0.28	0.2
20	Clostridia_uc_f	0.16	0.19	0.25
21	Erysipelotrichaceae	0.22	0.24	0.1
22	Elusimicrobiaceae	0.11	0.44	0
23	DQ809955_f	0.1	0.05	0.26
24	AF371945_f	0.01	0.11	0.28
25	Lactobacillaceae	0.04	0.22	0.11
26	AM275436_f	0.04	0.1	0.2
27	Bacteroidia_uc_f	0.09	0.12	0.13
28	Pasteurellaceae	0.11	0.09	0.11
29	Erysipelotrichi_uc_f	0.03	0.05	0.22
30	EF445272_f	0.02	0.01	0.19
31	Peptostreptococcaceae	0.02	0.06	0.09
32	DQ809526_f	0.02	0.04	0.1
33	Clostridiaceae	0.04	0.06	0.06
34	GU112192_f	0.01	0.02	0.11
35	AM275436_o_uc	0.02	0.01	0.07
36	EU844239_f	0.01	0	0.09
37	Mogibacterium_f	0.01	0.03	0.04
38	Enterococcaceae	0	0.07	0
39	Burkholderiales_uc	0.04	0.03	0
40	AB185535_f	0.06	0.01	0

Table S8. The comparison of the bacterial species differentially abundant in the the gut microbiome in Ctrl and EX groups by DESeq2 differential abundance analysis. *p* value < 0.05 is represent in the table.

	Species	baseMean	log2FoldChange	lfcSE	stat	pvalue
2	*Bacteroides fragilis*	7.712	-5.918	1.757	-3.368	0.001
3	*Phascolarctobacterium faecium*	6.37	-5.788	2.021	-2.865	0.004
4	*Megasphaera elsdenii*	28.748	-5.538	2.052	-2.699	0.007
5	*Megasphaera_uc*	5.9	-4.627	2.152	-2.15	0.032
6	*Streptococcus parasanguinis*	1.646	-4.411	2.185	-2.019	0.043
7	*DQ793660_s*	2.672	-4.134	1.687	-2.45	0.014
8	*Veillonella dispar*	4.824	-4.127	1.467	-2.813	0.005
9	*Bacteroides caccae*	7.104	-3.796	1.006	-3.773	0
10	*Eubacterium siraeum*	1.692	-3.515	1.288	-2.729	0.006
11	*DQ805681_s*	11.683	-3.475	0.906	-3.836	0
12	*4P000072_s*	4.128	-3.473	1.301	-2.669	0.008
13	*DQ456272_s*	2.733	-3.235	1.389	-2.33	0.02
14	*EU777390_g_uc*	3.142	-3.206	1.197	-2.677	0.007
15	*Parabacteroides distasonis*	2.533	-3.118	1.209	-2.58	0.01
16	*DQ905718_s*	0.607	-2.993	1.336	-2.24	0.025
17	*EF404287_s*	8.326	-2.932	1.027	-2.854	0.004
1	*Bacteroides vulgatus*	129.563	-2.799	0.699	-4.003	0
18	*EF401882_g_uc*	2.829	-2.603	1.277	-2.038	0.042
19	*DQ804825_s*	3.54	-2.538	1.055	-2.406	0.016
20	*DQ905034_g_uc*	5.813	-2.481	0.881	-2.814	0.005
21	*Odoribacter_uc*	0.802	-2.405	1.214	-1.981	0.048
22	*EF404077_s*	7.992	-2.328	0.965	-2.413	0.016
23	*4P000883_s*	1.147	-2.104	1.07	-1.966	0.049
24	*Bacteroides uniformis*	19.496	-1.763	0.812	-2.172	0.03
25	*Veillonellaceae_uc_s*	34.798	-1.428	0.591	-2.415	0.016
26	*Clostridiales_uc_s*	61.756	-0.857	0.345	-2.481	0.013
27	*EF402172_s*	59.013	1.102	0.471	2.338	0.019
28	*Subdoligranulum_uc*	6.311	1.378	0.695	1.981	0.048
29	*Sutterella_f_uc_s*	2.33	1.866	0.853	2.187	0.029
30	*Bifidobacterium_uc*	23.13	2.084	0.996	2.093	0.036
31	*Sutterella_uc*	5.672	2.348	0.88	2.668	0.008
32	*GQ897158_s*	48.592	2.437	0.901	2.703	0.007
33	*Blautia_uc*	3.879	2.585	0.833	3.103	0.002
34	*Burkholderiales_uc_s*	1.598	2.73	1.216	2.245	0.025
35	*EU462041_s*	59.173	2.788	1.294	2.155	0.031
36	*EU461711_s*	57.319	3.412	1.521	2.243	0.025
37	*DQ795906_s*	17.478	4.572	1.879	2.433	0.015
38	*Dialister succinatiphilus*	8.913	4.73	2.267	2.087	0.037
39	*4P000023_s*	16.634	5.145	2.473	2.081	0.037

Table S9. The comparison of the bacterial species differentially abundant in the the gut microbiome in Ctrl and DS groups by DESeq2 differential abundance analysis. p value < 0.05 is represent in the table.

	Species	baseMean	log2FoldChange	lfcSE	stat	pvalue
1	*Blautia_uc*	3.879	2.720	0.774	3.512	0.000
2	*Bacteroides vulgatus*	129.563	-2.361	0.686	-3.443	0.001
3	*DQ795906_s*	17.478	6.378	1.856	3.436	0.001
4	*Bacteroides fragilis*	7.712	-5.054	1.726	-2.928	0.003
5	*EF402172_s*	59.013	1.258	0.459	2.742	0.006
6	*4P000072_s*	4.128	-3.474	1.270	-2.736	0.006
7	*DQ805681_s*	11.683	-2.412	0.890	-2.710	0.007
8	*4P000873_s*	4.106	-2.904	1.177	-2.467	0.014
9	*Megasphaera elsdenii*	28.748	-4.963	2.014	-2.464	0.014
10	*EF401818_s*	1.481	2.887	1.200	2.405	0.016
11	*4P000883_s*	1.147	-2.332	1.011	-2.307	0.021
12	*Bacteroides_uc*	188.848	-0.969	0.425	-2.282	0.022
13	*EF401233_s*	2.098	2.463	1.081	2.279	0.023
14	*Odoribacter_uc*	0.802	-2.625	1.156	-2.271	0.023
15	*Coprococcus_uc*	2.296	2.470	1.097	2.252	0.024
16	*Phascolarctobacterium faecium*	6.370	-4.250	1.987	-2.139	0.032
17	*Odoribacter_f_uc_s*	1.845	-1.567	0.734	-2.134	0.033
18	*Bacteroidaceae_uc_s*	41.825	-0.661	0.333	-1.988	0.047

Table S10. The comparison of the bacterial species differentially abundant in the the gut microbiome in EX and DS groups by DESeq2 differential abundance analysis. p value < 0.05 is represent in the table.

	Species	baseMean	log2FoldChange	lfcSE	stat	pvalue
1	*Veillonella dispar*	4.820	-4.720	1.460	-3.230	0.000
2	*EF401233_s*	2.100	-3.970	1.080	-3.690	0.000
3	*EF404193_s*	0.660	-3.470	1.740	-2.000	0.050
4	*Veillonella_uc*	0.770	-3.360	1.610	-2.090	0.040
5	*Bacteroides caccae*	7.100	-3.310	0.980	-3.370	0.000
6	*Parabacteroides distasonis*	2.530	-3.180	1.190	-2.670	0.010
7	*EF404287_s*	8.330	-2.990	1.020	-2.930	0.000
8	*EF404077_s*	7.990	-2.830	0.970	-2.920	0.000
9	*EF401818_s*	1.480	-2.600	1.240	-2.100	0.040
10	*Coprococcus_uc*	2.300	-2.420	1.130	-2.150	0.030
11	*EU777390_g_uc*	3.140	-2.390	1.160	-2.060	0.040
12	*Roseburia hominis*	1.550	-2.320	1.080	-2.150	0.030
13	*Eubacterium hallii*	1.010	-2.200	0.920	-2.400	0.020
14	*EF404388_s*	121.030	1.820	0.850	2.140	0.030
15	*GQ897158_s*	48.590	1.910	0.900	2.120	0.030
16	*Sutterella_f_uc_s*	2.330	2.180	0.840	2.600	0.010
17	*Bifidobacterium_uc*	23.130	2.190	1.000	2.200	0.030
18	*Sutterella_uc*	5.670	2.290	0.880	2.610	0.010
19	*4P000873_s*	4.110	2.800	1.210	2.300	0.020
20	*4P000023_s*	16.630	5.150	2.470	2.080	0.040
21	*Dialister succinatiphilus*	8.910	5.660	2.260	2.500	0.010

Table S11. The List of microbial species constituting more than 0.5 % in the gut microbiome of the three experimental groups. The Ctrl, EX, and DS represents the control, exercise, and diet shift groups each. The species has below 0.5% relative abundance after combining all three groups are not included.

	Species	Ctrl (%)	DS (%)	EX (%)
1	*Prevotella copri*	15.63	16.47	11.94
2	*Prevotella_uc*	5.96	5.93	3.6
3	*EU461603_s*	5.94	2.88	3.55
4	*Bifidobacterium pseudocatenulatum*	6.44	3.81	0.33
5	*Bacteroides_uc*	1.8	3.96	4.42
6	*Bacteroides vulgatus*	0.42	2.45	4.86
7	*EU462076_s*	4.48	1.35	1.76
8	*4P000745_s*	3.04	2.36	2.13
9	*EU475206_s*	5.18	0.83	1.34
10	*EF404388_s*	1.91	2.77	1.14
11	*Prevotella stercorea*	1.37	2.24	1.55
12	*Bifidobacterium adolescentis*	0.53	1.91	2.64
13	*Ruminococcaceae_uc_s*	1.27	1.36	2.23
14	*Clostridiales_uc_s*	1.02	1.39	1.95
15	*FJ363527_s*	1.67	1.04	1.44
16	*Bacteroides plebeius*	0.65	2.45	0.77
17	*Faecalibacterium_uc*	1.07	1.28	1.24
18	*Prevotellaceae_uc_s*	1.43	1.04	1.03
19	*Bacteroides coprocola*	0.29	2.21	0.86
20	*BAAX01003885_s*	0.61	2.19	0.39
21	*EF402172_s*	1.32	0.62	1.01
22	*Faecalibacterium prausnitzii*	0.86	0.67	1.32
23	*DQ797252_s*	1.62	0.63	0.52
24	*EU462041_s*	1.64	0.68	0.39
25	*Klebsiella pneumoniae*	2.02	0.08	0.48
26	*EU461711_s*	1.83	0.44	0.29
27	*Megamonas rupellensis*	0.87	0.08	1.53
28	*GQ897158_s*	1.09	0.84	0.32
29	*Bacteroidaceae_uc_s*	0.46	0.81	0.98
30	*Bacteroidales_uc_s*	0.65	0.77	0.82
31	*Lachnospiraceae_uc_s*	0.47	0.71	0.94
32	*Odoribacter_uc*	2	0.02	0.02
33	*Veillonellaceae_uc_s*	0.28	0.51	1.24
34	*Escherichia coli group*	0.14	1.31	0.53
35	*Megasphaera elsdenii*	0.2	0.55	1.2
36	*DQ801259_s*	0.52	1.1	0.31
37	*EF403870_s*	0.5	0.79	0.44
38	*GQ047204_s*	0.43	0.98	0.33
39	*Bacteroides eggerthii*	0	0.01	1.69
40	*DQ795058_s*	0.69	0.53	0.46
41	*Dialister succinatiphilus*	0.13	1.12	0.36
42	*Bifidobacterium stercoris*	0.26	1.07	0.3
43	*EU531928_s*	0.38	0.17	0.96
